# The Structure and Function of Next-Generation Gingival Graft Substitutes—A Perspective on Multilayer Electrospun Constructs with Consideration of Vascularization

**DOI:** 10.3390/ijms23095256

**Published:** 2022-05-09

**Authors:** Brian C. W. Webb, Michael Glogauer, J. Paul Santerre

**Affiliations:** 1 Faculty of Dentistry, University of Toronto, 124 Edward St, Toronto, ON M5G 1G6, Canada; b.webb@mail.utoronto.ca (B.C.W.W.); michael.glogauer@dentistry.utoronto.ca (M.G.); 2 Institute of Biomedical Engineering, University of Toronto, 164 Collage St Room 407, Toronto, ON M5S 3G9, Canada

**Keywords:** electrospinning, gingival tissue, material substitutes, functional materials, porous materials, vascularization

## Abstract

There is a shortage of suitable tissue-engineered solutions for gingival recession, a soft tissue defect of the oral cavity. Autologous tissue grafts lead to an increase in morbidity due to complications at the donor site. Although material substitutes are available on the market, their development is early, and work to produce more functional material substitutes is underway. The latter materials along with newly conceived tissue-engineered substitutes must maintain volumetric form over time and have advantageous mechanical and biological characteristics facilitating the regeneration of functional gingival tissue. This review conveys a comprehensive and timely perspective to provide insight towards future work in the field, by linking the structure (specifically multilayered systems) and function of electrospun material-based approaches for gingival tissue engineering and regeneration. Electrospun material composites are reviewed alongside existing commercial material substitutes’, looking at current advantages and disadvantages. The importance of implementing physiologically relevant degradation profiles and mechanical properties into the design of material substitutes is presented and discussed. Further, given that the broader tissue engineering field has moved towards the use of pre-seeded scaffolds, a review of promising cell options, for generating tissue-engineered autologous gingival grafts from electrospun scaffolds is presented and their potential utility and limitations are discussed.

## 1. Introduction

Gingival recession with tooth root exposure affects half of the adult U.S. population [1,2]. A more efficient and less painful solution to the current treatment standard could have a widespread impact, improving the lives of millions. Loss of gingival coverage around the tooth at the tooth–tissue margin is referred to as gingival recession and results in the exposure of the tooth’s root surface. This root exposure can lead to tooth sensitivity when eating, increased risk of biofilm accumulation and further tissue loss and aesthetic compromise. Tissue loss is primarily caused by inflammation associated with periodontitis (initiated from agents produced within plaque/biofilm) and mechanical trauma [3]. Not only does gingival recession yield challenges for the patients’ esthetic appearance, but it can also expose the roots surface of the tooth to cariogenic supragingival microbiota leading to an increased risk of dental caries and in the extreme case loss of tooth [3]. 

The current treatment for the soft tissue defect of gingival recession is primarily autologous soft tissue grafts, usually harvested from the patient’s palate [4]. However, material substitutes can be used in isolation, or with autologous grafts, and are available on the market, such as the Geistlich Fibro-Gide^®^ bovine-collagen-based material [5]. This material still has limitations when compared to the gold standard of care (autologous grafts) [6], while several other more innovative materials that are now being studied and are discussed here. However, the field of tissue material substitutes [7], and tissue-engineered solutions is still in its infancy in this application area. The pain and length of recovery and the time to carry out the procedures could be greatly reduced, when compared to the standard-of-care-associated procedures, if superior scaffold material substitutes and/or pre-vascularized tissue-engineered constructs could be translated into the clinical realm [8,9]. Vascularized tissue-engineered substitutes hold the potential to provide the cells needed for tissue regeneration and anastomosis, and deliver novel scaffolding materials to promote their proliferation and phenotype expression towards successful tissue regeneration outcomes [10].

One promising processing method for fabricating materials for regenerating and/or engineering gingival tissue is electrospinning. The method enables the production of fiber and fibril features that are on the scale of those of host extracellular matrices (ECM). Despite its mention in a recent systematic review looking at engineering vascularized oral tissue (mainly gingiva and alveolar bone), the article provided no insight into the use of layered electrospun scaffolds, which is gaining interest by many tissue engineering groups attempting to replicate the ECM form and niche residence conditions for related cells to the tissue being grown [11]. It should be noted that while other examples of layered scaffolds for periodontal regeneration have been previously reported, none have addressed the potential use of electrospun elastomeric polymers [7]. Thus, the goal of the current review is to provide focused insight on understanding the relationship between structure and function applied to new innovative electrospun-material-based approaches for gingival tissue engineering and regeneration. Specifically, functional electrospun materials are discussed in addition to a method for generating 3D electrospun constructs and providing perspectives on promising cell options for engineering pre-vascularized gingival tissue.

## 2. Physiology and Disease of the Periodontium and Gingival Tissues: Defining Structure Requirements

**Figure 1:**
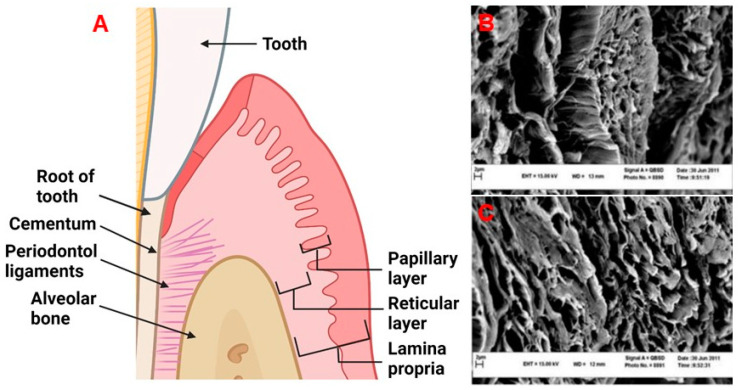
(**A**) The periodontal tissue anatomy. Created with BioRender.com. (**B**,**C**) Decellularized human gingival tissue adapted from previous literature reproduced under terms of the CC-BY license [16]. Copyright 2012, Nasser Mahdavishahri, Maryam Moghatam Matin, Masoud Fereidoni, Zahra Yarjanli, Seyed Ali Banihashem Rad, and Saeedeh Khajeh Ahmadi, published by Iranian Journal of Basic Medical Sciences. Created with BioRender.com, accessed on 8 April 2022.

The periodontium is comprised of four main tissue types: the alveolar bone, periodontal ligaments (connective tissue which allows for the attachment between the alveolar bone and root of the tooth), cementum, which is a mineralized tissue connecting the alveolar bone and the root of the tooth via periodontal ligaments, and gingival tissue which is the mucosal tissue that seals and protects the tooth from bacterial or physical threats as illustrated in Figure 1A [12]. The gingiva has two distinct layers, the epithelial tissue layer and the connective tissue layer (lamina propria) which make up approximately 30% and 70% of the gingiva, respectively [13,14]. The lamina propria can further be described as having two layers, the papillary layer, and the reticular layer [14]. The recession of gingival tissue is primarily caused by prolonged inflammation of periodontal tissue, periodontal treatment, and occlusal trauma [15]. Factors that could predispose an individual to gingival recession include a decrease in the thickness of the alveolar or buccal bone [15].

Healthy gingiva is comprised mostly of collagens [17], elastin [18], laminin [13], and fibronectin [13,19]. Of the collagens found in the gingiva, collagen type I and type III make up 99% of this protein family in human gingival tissue [17]. The remaining 1% is accounted for by collagen type IV, with the presence of collagen type V only increasing during the initial stages of healing. The presence of collagen type V is thought to guide endothelial cells (ECs), facilitating angiogenesis [17]. The major function of the remaining collagen molecules is primarily to provide strength to the lamina propria [17]. The ultra-structure of these collagens and ECM can be found in Figure 1B,C. Within the lamina propria, human gingival fibroblasts (HGFs) are responsible for synthesizing and maintaining the ECM [20]. Gingival fibroblasts are present in the lamina propria at a concentration of 200 million cells/cm^3^ [14]. The collagen fibrils produced by HGFs are approximately 50–100 nm in diameter [21]. Both the papillary and reticular components contain a dense network of vasculature, consisting of terminal capillary loops in the papillary component and the gingival plexus which is made up of postcapillary venules [14]. The papillary layer contains approximately 50–60 loops/mm^2^ [14]. The natural gingiva has approximately 10 microvessel lumens/mm^2^ [22,23], with defined diameters depending on locations and depth as outlined in Table 1. 

Having the blueprint of the vasculature within gingival tissue is fundamental to its engineering from the standpoint of understanding what is required for the native tissue to function. There are however notable differences in vasculature structure from person to person [25]. How and to what variation blood flow is being supplied to the lamina propria is of relevance when considering how important the anastomosis of pre-vascularized constructs will be. By prohibiting blood flow from certain areas of the papilla, previous literature has demonstrated that some individuals have greater blood flow horizontally or vertically [25]. This is thought to be related to the abundance of blood vessels supplying the gingival area [25]. The differences may also be explained by changes in arteriole-to-arteriole connections (<100 µm in diameter) [24,25,26]. It is also well recognized that males have better blood flow recovery, and quicker anastomosis of coronally advanced flaps than females, which may suggest key factors that can be targeted to enhance the anastomosis of a graft [27]. Characterizing the differences in gingival vasculature is ongoing and will be critical to the application of pre-vascularized tissue engineering to the periodontium. Based on the physiology and anatomy of gingival tissue, it is evident that to prepare a construct resembling the native gingiva, modulating vasculature formation is going to be critical to graft integration and healing. 

## 3. Current Material Options for Gingival Recession Treatment

**Figure 2:**
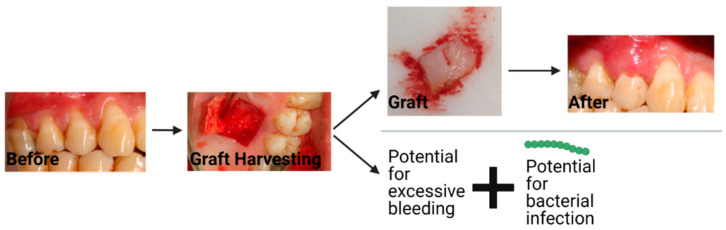
The workflow and potential complications that can occur with a connective tissue graft. The string of green dots represents bacteria. Created with BioRender.com with images from Dr. Michael Glogauer (University of Toronto) and images reproduced with permission under terms of the CC-BY license [31]. Copyright 2014, Sakshee Trivedi, Neeta Bhavsar, Kirti Dulani, and Rahul Trivedi published by the Journal of Clinical and Experimental Dentistry. Created with BioRender.com, accessed on 8 April 2022.

The current treatment for gingival recession is typically autologous soft tissue grafts [4]. Additionally, material substitutes are available on the market, which have some reports on their efficacy. The two most common types of autologous grafts are connective tissue grafts (CTG) and free gingival grafts (FGG). CTGs involve harvesting connective tissue and grafting it such that root coverage and improved thickness of the gingival tissue are provided, as seen in Figure 2. An FGG entails harvesting connective tissue with surface epithelial tissue and placing it on the defect to cover the exposed root of the tooth and increase keratinized tissue [28]. Some of the major disadvantages of autologous gingival grafting are the increase in morbidity due to the harvest site, interindividual differences in terms of tissue availability, the time associated with the tissue harvesting (FGG takes ~25 min longer than using material substitutes) [29], donor infection, and bleeding from the harvest site [5,30]. To avoid some of the downsides of autologous grafting, material substitutes can and have been introduced in an attempt to address these issues, however, the standard of care remains the autologous graft, which highlights the limitations of the current alternatives, some of which will be discussed later in this review.

Based on two systematic reviews, the general consensus is that FGGs provide additional efficacy in terms of generating keratinized tissue when compared to material substitutes [6,32]. One of the most popular metrics of efficacy is the width of keratinized tissue [32]. Although the differences between autografts and material substitutes might not be large enough to negate the use of material substitutes given their benefits, autologous grafts remain the “gold standard” due to their ability to provide a greater area of keratinized tissue [6,32]. Taken all together autologous grafts remain the engineering benchmark, in large part due to their superior efficacy quantified by keratinized tissue. 

During the differentiation of epithelial cells to keratinocytes, the composition of the underlying connective tissue dictates the subsequent occupancy of the epithelium, due to the keratinocyte interactions with components of the ECM, such as collagen type I [33,34]. Keratinocytes are also involved in the complex processes of healing the underlying connective tissue [35]. Further, changes in epithelial cell integrin binding are associated with changes in the activity of matrix metalloproteinases (MMPs), which participate in tissue remodeling and the migration of keratinocytes [33,36]. The interactions between the underlying connective tissue and the epithelial layer are mediated by integrins such as beta1-integrins [37]. The interactions that epithelial cells, such as keratinocytes, have with such integrins dictate cellular differentiation and survival [37,38]. Additionally, the secretion of paracrine factors such as hepatocyte growth factor from the underlying connective tissue contributes to the formation of keratinocytes [39]. 

Due to the available supply and lower morbidity associated with using material substitutes, the field is pushing towards their greater adoption. However, their adoption is in part hampered by the lack of efficacy to establish a keratinized structured layer, as discussed above, when compared to the traditional autologous graft. In considering keratinized tissue dependence in relation to their composition and interactions with the underlying connective tissue, it will be important that de novo material substitutes try to facilitate the formation of healthy gingival tissue more rapidly, as in this instance more keratinized tissue can be produced, thus improving the clinical efficacy of the graft. 

Currently, the most common types of material substitutes being reported on and used in the clinic appear to be xenogenic scaffolds such as the Geistlich Fibro-Gide^®^ [5], or allogenic grafts such as Alloderm^®^ [40,41]. Their benefits mainly reflect their unlimited supply relative to autografts and lower associated morbidity relative to other material subsitutes [42]. Geistlich Fibro-Gide^®^ by volume is 96% porous and is comprised of 60–96% (*w*/*w*) porcine collagen (type I and III) and 4–40% (*w*/*w*) elastin [43,44]. A similar product is Mucograft^®^, which has two layers, one of which is compact, and one which is spongy architecture for supporting cell ingrowth [43]. The compact outer layer can be left exposed to the oral environment and can aid in gaining keratinized tissue, suggesting that having a multilayered structure wherein different layers provide different functions is being used in the clinic [43]. Mucoderm^®^ is a similar product to Mucograft^®^ however is only comprised of a single layer [43]. Alloderm^®^ or decellularized human dermal tissue are used clinically, however, they have been shown to have inferior clinical outcomes when compared to FGGs [45,46,47]. These material substitutes are currently the leading commercial substitutes that are widely available but are clearly still in need of improvement [5]. These material substitutes lack many of the features discussed early in this review and that would be essential for successful grafting and define the field as being in its infancy. 

## 4. Electrospinning Biomaterial Features for Gingival Tissue Engineering

**Figure 3:**
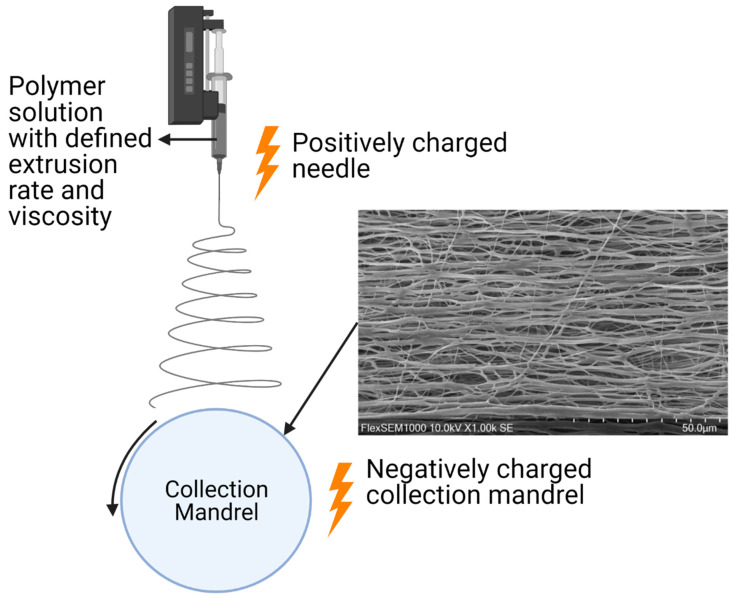
The electrospinning process with a scanning electron microscopy image of an electrospun scaffold taken by the author. Created with BioRender.com, accessed on 8 April 2022.

Electrospun scaffolds offer several of the characteristics necessary to foster and modulate soft tissue regeneration [48], while closely resembling the physical fiber features of native ECM [49]. Electrospinning facilitates the formation of fibers/fibrils by extruding a polymer solution through an electrostatic field that is generated through a capillary needle with a high voltage that is met by a grounded collection mandrel at a defined distance [50]. Fibers between a few nanometers and greater than 1 µm can be formed using this process [50,51,52]. With a multitude of parameters (illustrated in Figure 3) influencing the electrospinning process such as voltage, the polymeric solution properties, and flow rate, the electrospinning process almost always requires optimization of parameters when one material is changed to the next. It is also these fabrication parameters that allow for diverse scaffold morphologies to be achieved [53]. Lastly, the electrospinning process is rapid and cost-effective [49]. Specifically, it does not require extensive purification steps, enables low-cost processing steps, and reduces production time. The latter overcomes several shortcomings associated with obtaining decellularized tissue for example.

The range in fiber membrane properties that can be achieved allows for the application of this process to produce fibers for engineering different tissue types. As discussed in a previous review the phenotype of cells seeded on electrospun scaffolds can be modulated by altering the defined nanotopography of the fiber membranes [48]. Gene expression involved in cellular behavior and signaling pathways can even be modulated by altering factors such as pore size [54]. 

In a 2009 study [55], the authors reported on a paradigm shift from the use of porous foams towards electrospun fibers for guided periodontal regeneration; however, this was not discussed specifically in the context of gingival tissue and its vascularization. The function of using aligned electrospun fibers for culturing HGFs for gingival tissue engineering has been presented [56]. The addition of cells offers promising phenotypic character to enhance the function of electrospun scaffolds for gingival tissue engineering. Of relevance to gingival tissue specifically, an increase in the production of collagen type I has previously been shown to be achieved with HGFs on aligned versus random fibers [57]. With collagen being the main component of the lamina propria’s ECM [17], using an aligned fiber may offer a convenient alternative to begin engineering tissue that could be more representative of the native tissue. Other authors investigating fiber alignment in the context of gingival tissue regeneration found an increase in HGF proliferation, collagen type I, focal adhesion kinase, and fibronectin on aligned versus random electrospun poly-ε-Caprolactone (PCL) scaffolds [56]. Although in this example PCL fibers were used, the benefit of using aligned fibers could be applied to electrospinning any material. This suggests that based on alignment only, the cells seeded on aligned electrospun scaffolds may offer greater gingival fibroblast proliferation, and collagen type I production when compared to non-aligned material substitutes. Interestingly, the Geistlich collagen-based scaffolds (e.g., Fibro-Gide^®^, Mucograft^®^, and Mucoderm^®^) currently on the market have a randomly aligned structure, suggesting that their function could be improved.

When evaluating material substitutes as potential grafting materials, pore size and percent porosity are critical considerations as they dictate factors such as cellular and vascular ingrowth, and transportation of oxygen, waste, and nutrients [58]. Pore size has previously been altered/tuned by using sacrificial polymers which are initially included in the electrospinning process and then washed away with water [59,60]. Small pore sizes can inhibit vascular ingrowth (needed for the diffusion of nutrients, oxygen, and waste) [50]. A general range of pore size which has been shown to allow for cellular infiltration is in the range of 100–500 µm [61,62]. Electrospun poly-L-lactic acid (PLLA) scaffolds have previously been fabricated for skin tissue engineering with an average pore size of 132.7 µm and porosity of ~92%, with the higher porosity scaffolds showing an increase in cell migration, infiltration, and collagen deposition [58]. The high porosity and tissue infiltration are consistent with the Geistlich scaffolds (e.g., Fibro-Gide^®^, Mucograft^®^, and Mucoderm^®^) which have a porosity of ~93% by volume [44]. 

Electrospinning facilitates the formation of fibers with diameters in the same range as that of the collagen and other relevant supporting fibers such as elastin. Collagen type I and other fibril forming collagen fibrils have a diameter ranging from ~25–400 nm [63]. Elastin fibers and fibrils have diameters of 1 µm and 0.2 µm, respectively [64]. These are well within the diameter range of what can be electrospun [50,51,52]. The underlying importance and significance of having fiber diameters recapitulating those observed in native tissue are complex and likely reliant on if the material is intended to be pre-seeded with cells or be used as a substitute acellular material. Further, the optimal characteristics of the material may be different depending on if the material is intended to be pre-seeded with cells or be used as a substitute acellular material, however, a direct comparison is still needed. For example, previously it has been concluded that a larger fiber diameter (4.83 µm versus fiber diameters ranging from 1.64–3.37 µm) resulted in human umbilical vein endothelial cells (HUVECs) having significantly greater scaffold infiltration, viability, and CD31 expression [53]. Another study seeded vascular smooth muscle cells (VSMC) on PCL fiber membranes with varying diameters (0.5, 0.7, 1, 2, 2.5, 5, 7, and 10 µm) and concluded that a larger diameter (7 and 10 µm) allowed for greater VSMC and macrophage infiltration when compared to the lower fiber diameter scaffolds [65]. One paper published in 2021 supported that the cellular phenotype of seeded cells is modulated by the fibers’ electrical charge (piezoelectric properties) which were also shown to be influenced by fiber diameter [66]. A previous review has covered recent literature regarding how different electrospun fiber characteristics affect immune response [67]. Although the literature offers a range of porosities and fiber diameters that influence cellular phenotype, there still appears to be no defined standard for gingival tissue engineering. 

When comparing the current relative benchmark grafting materials structure to electrospun materials, we do see similarities in structure. It is no surprise that Geistlich scaffolds (Fibro-Gide’s^®^, Mucograft^®^, and Mucoderm^®^) structure resembles the collagen fibrils found in the native gingiva because it is made from bovine-derived collagen. In addition to having a fiber structure that resembles the native tissue (similar to the Geistlich materials), electrospun materials allow for many other factors to be controlled (e.g., porosity, fiber diameter, etc.) [49]. The scientific field appears to be moving towards materials with much more function and modulatory features, targeted at enhancing tissue development, and tissue engraftment beyond what is offered by a simple collagen scaffold. 

## 5. Layered Structures in Electrospun Constructs

It is well established that electrospun scaffolds can be designed to closely resemble that of the native ECM [49]; however, using a single layer electrospun membrane does not enable the engineering or regeneration of tissues with considerable thicknesses and the appropriate defined cellular densities that are needed. Thus the use of layered electrospun scaffolds has become more evident in the literature [7]. The use of layered scaffolds for the broader concept of periodontal regeneration has been noted and reviewed by authors previously and described as a burgeoning concept [7]. However, the literature is relatively limited with respect to articles that have reviewed the use of layered electrospun scaffolds, specifically for regenerating and engineering gingival tissue. Given the tissue-specific dependence of tissue engineering methods on the different cell phenotypes, vascular bed density generated, and ECM composition/production as discussed above, it is relevant to provide the field with particular attention to such design considerations. 

**Figure 4:**
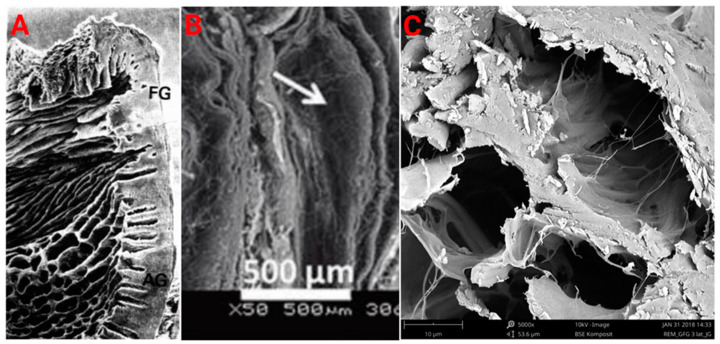
Scanning electron microscopy images of different gingival tissue and previously reported constructs. (**A**) Scanning electron microscopy images of native gingival tissue. FG: free gingival, AG: attached gingiva. Reproduced with permission [68]. Copyright 1981, published by the Journal of Periodontal Research. (**B**) Scanning electron microscopy images of layered poly(l-lactic acid) electrospun scaffolds with adipose-derived stem cells and collagen. The white arrow indicates delamination of the scaffold layers. Reproduced with permission [70]. Copyright 2010, published by the Tissue Engineering—Part C: Methods. (**C**) Scanning electron microscopy images of a Geistlich^®^ Fibro-Gide^®^ matrix. Copyrights by courtesy of Geistlich Pharma AG.

Gingival tissue could be considered as having layers in both the horizontal (transverse) and vertical (longitudinal) axes. As illustrated in Figure 4A, the native gingiva consists of layers that are vertically stacked in the free gingiva, with these defined layers taking on a honeycomb structure in the attached gingiva [68]. Figure 4B illustrates layered electrospun scaffolds which have structural similarities to native gingival tissue. In the horizontal direction, gingival tissue is composed of three main layers, an epithelium composed of many layers of keratinocytes with complex and diverse functionality [7,69], the basement membrane, and the lamina propria which consists of a papillary and reticular layer containing gingival fibroblasts, vasculature, and collagen-rich ECM [13,14,17]. Therefore, it is supported that the use of layered electrospun scaffolds, which architecturally mimic the divergent aspects of the native gingiva, could be generated. 

### 5.1. Horizontal Layers

The complete biological function of tissue forming layers appears to be quite complex and likely is not fully understood. However, some specific examples can help us understand its potential relevance for gingival tissue engineering. The engineering or regeneration of full-thickness gingival tissue requires the consideration of three main tissue layers in the horizontal direction [13]. Most proximal to the alveolar bone or tooth, the stratified squamous epithelium must be present to protect the tissue from both bacterial and mechanical threats [71]. The next layer is the basement membrane which is critical in separating the lamina propria and epithelium [13]. The basement membrane consists of anchoring fibers, integrins, laminin, and collagen type IV, which are necessary for the attachment of cells in the lamina propria and epithelium [13]. Not only does the basement membrane dictate cellular attachment but is also involved with cellular differentiation and phenotype [72]. 

In an in vitro model, it has been understood that the behavior of epithelial cell layers (which function to protect the tooth from bacterial and mechanical threats) is modulated by/through adherens junctions, which facilitate the formation of epithelial cells into a layer above a sheet of fibroblasts [73]. Further, the connection between the epithelial cell sheet layer and the underlying fibroblast layer is important such that they are required to prevent apoptosis [74]. These adhesions between the epithelial and fibroblast layer play a role in mechanically stabilizing the cell sheet, cell migration, reorganization, and random cell movement [73,75]. Additionally, the keratinocytes, found within the epithelium of the gingiva, form layers themselves with functional differences [13,76]. Thus, engineering gingival tissue in sheets or layers may offer a method for recapitulating this interaction.

### 5.2. Vertical Layers

In the vertical or longitudinal direction within the lamina propria, we also observe layers of tissue. In vitro when gingival fibroblasts are grown on cementum they form sheets (qualitatively resembling an electrospun scaffold) [21,77,78]. Additionally, when fibroblasts are cultured in the presence of transforming growth factor-β they form sheets [79,80]. Transportation and diffusion of oxygen, nutrients, waste, and bioactive molecules are anticipated to be a function of the tissue layers. The movement of these molecules throughout a tissue is critical for tissue remodeling and regeneration. The characteristics of electrospun scaffolds such as porosity have exciting implications for engineering layered tissue, as these parameters could control the diffusion of bioactive molecules and can be optimized for factors such as cellular ingrowth, cellular proliferation, and diffusion of cellular waste, nutrients, and oxygen between layers [81]. Bovine-collagen-derived materials currently used, do not allow for the optimization of all these parameters.

A previous study showed that the layering of electrospun scaffolds (polycaprolactone and polycaprolactone/collagen), with the layers being seeded with either ECs or fibroblasts, facilitated the formation of vessels in an in vivo rat model, with red blood cells being found in the middle of a three-layer construct after just one week [82]. Interestingly, the formation of vasculature was dependent on the number of electrospun scaffold layers, and the presence of endothelial cell layers. This supports that layered electrospun scaffolds can facilitate the formation of vasculature in a way that would be expected to permit the movement of waste, oxygen, and nutrients, supporting tissue regeneration. In vivo after one week of implantation, red blood cells were found at the center of a three-layered construct. This study provides strong support that electrospun multilayered constructs seeded with ECs and fibroblasts provide an adequate environment for the formation of vasculature [82]. Considering that vasculature in the native gingiva is present (10 lumens/mm^2^ [22], with the diameters outlined in Table 1), potential material or tissue-engineered substitutes must support the formation of vasculature, which layered electrospun scaffolds have directly been shown to facilitate. More comprehensive pre-clinical animal models will be needed to evaluate multilayered constructs in a gingival tissue-specific in vivo environment.

To further improve the function of the layered electrospun scaffolds, micron-sized laser-cut ablations have previously been made through layers of an electrospun poly (l-lactic acid) scaffold, a cross-section of which can be seen in Figure 4B [70]. The approximately 300 µm in diameter laser-cut pores enhanced the proliferation, and viability of seeded human adipose-derived stem cells (ASCs), compared to non-ablated scaffolds [70]. Further, the ablations helped to prevent the separation of the scaffold layers and maintain the multilayered structure [70]. Collagen type I was also pipetted onto the scaffold before layering, to improve the attachment between the cell-seeded scaffold layers [70]. The use of laser-cut ablations may offer a method to decrease the separation of scaffold layers, and improve cell viability and proliferation [70]. 

The conclusion is that there is in vivo evidence for multilayered electrospun constructs facilitating the formation of vascularized tissue, but future work should continue to pursue the use of layered electrospun scaffolds for specifically gingival tissue engineering, as an alternative to the Fibro-Gide^®^, Mucograft^®^, and Mucoderm^®^ materials. Vertical and horizontal gingival tissue-engineered layers could later be combined, to closely recapitulate the native gingival tissue. Although layering electrospun materials appears to provide a potentially viable architecture for gingival tissue engineering, another important consideration is the material itself. 

## 6. Conventional Material Approaches

There are three types of scaffolding materials that are relevant to the gingival tissue engineering application: natural polymers, synthetic polymers, and hybrid/composites [83]. All three of these latter groups are most appropriate and popular for soft tissue engineering due to their ability to recapitulate several physical/biochemical aspects, function, and architecture of the native tissue [84]. 

In general, natural scaffolds have fast and inconsistent degradation with poor mechanical properties [85,86]. Specifically, the limitations of the collagen matrices are that degradation and tissue integration must be balanced with mechanical properties. As the degree of crosslinking is increased within the matrix, there is also an increase in mechanical stability and degradation resistance, but tissue integration is decreased [43]. Many crosslinking methods have drawbacks which include inflammatory responses to the reagents and the foreign nature of the crosslinked structures [43,87]. Additionally, the proportion of collagen to elastin can be altered to change the scaffold’s mechanical properties and degradation (although degradation was not directly studied by the referenced authors) [88]. However, this is limited by the innate characteristics of the biomolecules used [88]. Fibro-Gide^®^ (seen in Figure 4C) has a porosity of ~93%, increases in volume by ~25% when wetted [43], and an elastic modulus of ~5.9 × 10^−3^ Mpa [89], with the elastic modulus of native gingival tissue being ~37.4 Mpa [90]. 

When the Fibro-Gide^®^ membranes are implanted in vivo, most of the material appears to degrade within 90 days, with some elastin still present thereafter [91]. Even after 90 days, there does appear to be tissue remodeling [91]. The use of decellularized human tissue or Alloderm^®^ for gingival tissue engineering has also been explored and used clinically, however, the material has limited cell infiltration, inconsistent degradation, and a high cost compared to their competition [40,41,45,46,47]. Thus, likely explains the material’s inferior clinical performance when compared to FGGs [45,46,47], and limited uptake by clinicians. 

Polyurethane-based materials have also been used clinically, such as Artelon^®^, a porous polycaprolactone-based polyurethane urea scaffold. One study showed the material can allow for a marketable improvement in volume for buccal soft tissue augmentation [92,93]. Few studies have investigated the use of this material for gingival tissue engineering, with the longest reported follow-up being only 6 months [92,94]. The potential downside to the material is its relatively slow degradation of approximately 6 years, which may hamper tissue integration and the formation of functional gingival tissue [92,93]. The potential concern with lacking functional tissue development is the inability for the required cellular interactions to occur between keratinized tissue and the underlying tissue [38]. 

Synthetic materials such as expanded polytetrafluorethylene (ePTFE), polylactic acid (PLA), and PLA-polyglycolic acid (PLA-PGA) have been evaluated clinically for treating gingival recession and previously reviewed [95]. The use of ePTFE has major drawbacks, as a second follow-up appointment is needed to remove the non-degradable material. When comparing ePTFE to PLA no significant difference was detected in terms of keratinized tissue 6 months post-surgery [95]. Further as reviewed previously [95], an alarming reaction, characterized by swelling and a large foreign body reaction including multinucleated giant cells, is associated with the use of PLA as a gingival grafting material. PGA [96], and the slower degrading poly(glycolide-co -L-lactic acid) or PGLA, have also been investigated for gingival tissue engineering applications [97,98]. PGLA has inferior fibroblast attachment and proliferation with poor epithelial morphogenesis when compared to natural scaffolds [85,99,100]. 

Compared to natural polymers synthetic scaffolds are generally considered to have superior mechanical properties, are more reproducible, and are more economical [101]. Pros and cons exist for both natural and synthetic scaffolds, however, the cons may be able to be negated by using a more complex approach that exploits the pros of each material type to negate their respective cons. 

## 7. Current Direction in Material Development

The criteria and future directions of material substitutes for gingival tissue engineering are that they be: non-infectious, biocompatible, allow for rapid tissue integration, facilitate the efficient formation of vascularization, maintain volumetric form over time, have mechanical characteristics that allow for practical clinical handling, and are economical [102]. Although all design features may be improved by using novel material substitutes, the area that likely warrants the greatest improvement is the time towards tissue integration and vascularization, while not compromising mechanical stability. 

To overcome some of the challenges and improve upon the conventional material approaches the development of new materials for gingival tissue engineering appears to be leaning towards the use of blended or composite/hybrid biomaterials, constituting multiple natural and/or synthetic polymers, with or without added bioactive molecules. This allows for a scaffold with greater function. While the use of composites has been previously alluded to in relation to the use of electrospun polymer blends for fabricating more functional scaffolds [103,104], the approach applied to the specific context of gingival tissue engineering is lacking in the literature. Of particular relevance, are the unique criteria that are needed for gingival tissue engineering which might be best met by using blended or composite biomaterials.

Previously the use of such hybrid scaffolds has allowed for the control and tuning of a scaffold’s relevant physical properties. A blend of gelatin and PLA was electrospun and characterized [105]. The combination of natural and synthetic polymers facilitated the fabrication of scaffolds with unique fiber diameters, hydrophilicities, and porosities; this could be optimized for gingival tissue engineering. The combination of a synthetic material such as PLA, which is hydrophobic, slowly degrading, and has good mechanical properties can be well complemented with a hydrophilic natural-based material, which has excellent cell adhesion, has timely degradation, but limited mechanical properties (e.g., gelatin). Essentially the materials address each other’s downsides, resulting in a more optimal and functional material for gingival tissue engineering. Although the example of the PLA/gelatin scaffold [105], and a previous review on electrospun polymer blends for fabricating more functional scaffolds were looked at through a broader tissue engineering lens [103,104], the underlying fundamental relation between the structure and function of the electrospun fiber materials also has promise for engineering gingival tissue material substitutes. 

In 2019, an electrospun scaffold with varying ratios of PCL and dicalcium phosphate dihydrate (DCPD) was investigated for bone tissue engineering [106]. The addition of the DCPD structure to the PCL scaffold improved the material’s hydrophilicity and fluid absorption in addition to improving the cell viability of HGFs, a relevant cell type for gingival tissue engineering [106]. The improvement in function is likely explained by the increase in surface roughness, changes in fiber diameter, and hydrophilic properties [106].

Poly(vinyl alcohol) and sodium alginate (PVA/SA) scaffolds have previously been electrospun at 10 wt% and 3.5 wt%, 4 wt% and 5 wt%, respectively [107]. The growth of HGFs on each scaffold was then investigated [107]. The structure provided by using a 4 wt% PVA/SA solution was determined to have the highest biocompatibility with electrochemical properties, suggesting that mature cell interactions were occurring between HGFs [107]. The authors suggested that it was the function provided by the dielectric properties specific to the 4 wt% PVA/SA scaffold that is enabling a scaffold with improved biocompatibility, determined by a greater HGF density, and coverage of the scaffold [107]. Cons to the use of PVA are that it is non-hydrolyzable, and has a history of being a complement system activator [108], which can negatively impact wound healing [109].

Previously aligned PCL, basic fibroblast growth factor (bFGF)-loaded electrospun membranes coated with self-polymerized dopamine conjugated with heparin, have been investigated as a material substitute for gingival tissue grafting [110]. In this study, seeded NIH-3T3 cells adhesion, and adhesion morphology was improved in the coated and loaded group, with a synergistic effect being detected in fibroblast proliferation in the aligned, coated, and loaded group [110]. The addition of a polydopamine coating and heparin immobilization changed the water contact angle of the material from 120° to 30°, essentially making a hydrophobic scaffold hydrophilic [110]. The combination of both a bioactive molecule (bFGF) and fiber alignment is reported to synergistically enhance tissue regeneration [110]. This is likely due to the aligned fibers, and the presence of bFGF recapitulating structural and molecular (bioactive molecules) factors that are seen in native tissue. This is a great example of how the field is pushing to generate more functional material substitutes when compared to the currently used Fibro-Gide^®^, Mucograft^®^, and Mucoderm^®^ materials.

To improve cellular/tissue infiltration sacrificial polymers have previously been used in the electrospinning process. Previously, a polyvinylpyrrolidone (PVP) and collagen solution were co-electrospun with poly(L-lactide-co-ε-caprolactone) (PLLCL). Post-electrospinning the PVP was removed by rinsing with water [59]. In another example, poly(desamino tyrosyl-tyrosine carbonate) (PDTEC) was electrospun with poly(ethylene glycol) (PEG) [60]. The PEG was used as a sacrificial polymer which once removed, increased the porosity of the scaffold facilitating the infiltration of cells [60]. This approach offers another potential strategy to modulate mechanical properties, degradation, and cellular infiltration.

A number of electrospun fibers with antibacterial properties have also been previously prepared [111,112]. Some of the antibacterial agents that have previously been introduced into electrospun scaffolds for periodontal engineering are bismuth subsalicylate [113], ampicillin [111], and ciprofloxacin-based additives [114]. The current relative benchmark grafting materials do not explicitly use antibacterial additives, suggesting that the incorporation of antibacterial molecules into material substitutes may be advantageous. Although, the actual rate of infection with scaffolds such as Fibro-Gide^®^ appears to be lacking from the literature. Thus, making it difficult to evaluate what impact the use of antimicrobial biomaterials could have. Further, if infection did occur this can be treated with oral antibiotics.

Silk is a material that has previously been used for oral mucosal tissue engineering [115,116]. One example which exemplifies the use of multi-functional electrospun scaffolds for oral mucosa regeneration was published in 2020. Silk electrospun fibers were modified with the addition of surface-aminated liposomes which were encapsulating leptin (NH_2_-LIPs) [116]. Polydopamine (PDA) was also synthesized onto the surface of the silk fibers. The catechol groups on the PDA can then react with the amino groups on the NH_2_-LIP, facilitating the fabrication of a silk fiber modified with PDA and NH_2_-LIP [116]. The addition of PDA and/or NH_2_-LIP led to the water contact angle of the material falling from 64 degrees to ~0 degrees [116]. The leachate from the scaffold with PDA and NH_2_-LIP resulted in human umbilical vein endothelial cells (HUVECs) forming a greater area of tubular structures or meshes after 10 h in culture, compared to the leachate of the non-functionalized silk fibers and silk fibers coated with only PDA [116]. In vivo, the electrospun scaffolds with PDA and NH_2_-LIP accelerated wound closure in an oral defect rabbit model. The scaffolds may be improved by using aligned electrospun fibers rather than random fiber arrangements.

The functionality of electrospun scaffolds for gingival tissue regeneration has previously been improved upon in a recent study (2021) where polycaprolactone (PCL) fibers were enriched with vitamin E and hyaluronic acid, and seeded with HGFs [117]. It was reported that HGFs seeded on the fibers with vitamin E and hyaluronic acid had statistically greater proliferation and gene expression, which induced a phenotype conducive to collagen deposition [117]. Vitamin E and hyaluronic acid inclusion may offer a relatively easy approach to enhancing cellular proliferation and gene expression of HGFs which could be implemented in future material substitutes.

Although the use of synthetic polymers such as PCL are popular in the literature, likely due to them being economical, well studied, and approved by the Food and Drug Administration (FDA), they have some major drawbacks. Specifically, PCL has limited recognition sites for cells, is hydrophobic [118], and has been shown to elicit a major immune response when used as a material substitute [95]. However, improvements are being made by producing composite materials, and thus, it is the latter compositions that include PCL that may have a place for gingival tissue engineering, rather than the PCL fibers themselves. One must consider that if the secondary composite components are leachable, then at some time the PCL will be left alone for an extended period of time given its slow degradation rate, and hence the above shortcomings re-emerge later in the implant life, which could be problematic. 

One potential limitation of using composite/blended polymer electrospinning is the necessity for a solvent that is suitable for electrospinning and solvates all the included polymers. This consideration has been previously reviewed [103] and may require modifications and optimization to be made for the electrospinning process of gingival tissue constructs.

### Degradation of Scaffold Materials and Mechanical Properties

When considering the optimization of materials for gingival tissue engineering, one major relationship of importance is the association between the mechanical properties of a material and the degradation of the material. We have seen both ends of this spectrum used, with synthetic-based materials having appropriate mechanical properties but relatively slow degradation, and natural materials such as Fibro-Gide^®^ (bovine-collagen-based) having relatively quick degradation and weak mechanical properties, but superior biocompatibility [43]. The approach of improving the mechanical properties of natural polymers by adding a synthetic material with superior mechanical characteristics has been extensively reported in previous literature [119].

The native gingival tissue has an elastic modulus and tensile strength that are approximately 37.4 MPa and 3.8 MPa, respectively [90]. However, human buccal mucosa, which has been used for autologous grafting to treat gingival recession, has an elastic modulus and tensile strength of approximately 8.3 MPa and 1.5 MPa, respectively, which was found to be significantly different than human gingival tissue [90]. Therefore, the mechanical properties of a tissue-engineered gingival graft could better reflect that of the native gingival tissue when compared to the current grafting tissue. This difference in mechanical properties suggests that a material substitute or engineered construct may only need to have a modulus within the range of what is seen in oral soft tissues.

The mechanical characteristics of a material for gingival tissue engineering become important when you consider the mechanical stressors that the material or engineered tissue would be subjected to; this occurs during or from speech, mastication, orthodontic movement, and during wound healing (e.g., blood flow and sutures) [120]. A bioreactor has previously been developed to screen potential cell-seeded material substitutes for oral soft tissue grafting [120]. The bioreactor determines and controls the shear force and pressure exerted on the cell-seeded material and allows for the subsequent observation of the material’s responses to different forces and pressures. This could be a potential tool for screening engineered gingival tissue constructs [120], however, a limitation is that there are still relatively minimal data available for quantifying the actual forces that occur in vivo [120].

Another mechanical property consideration of a potential material substitute for gingival tissue repair is the suture pull-out strength, as materials need to withstand the shear stress of a suture. The current standard for suture retention or pull-out strength is 2N, and would likely need to be a criterion for any material substitutes to be considered for translation [121,122].

When considering the degradation rate of a potential substitute material, generally the material needs to degrade fast enough for the host or seeded cells to integrate into the material. Though, slow enough to not leave the cells without a scaffold to adhere to, and for the graft to retain its volume. The use and clinical comparison of ePTFE (sold by Gore^®^) and a PLA membrane (sold by Guidor^®^) has previously been made in a canine model [123]. The non-resorbable ePTFE material does not degrade and must be removed after grafting, typically after 4–6 weeks. Thus, non-degradable materials are not intended for regenerating the gingiva through cellular infiltration, which would be required to form functional gingival tissue. The importance of regenerating the gingival tissue rather than only filling in the volume cannot be underscored enough, as the literature suggests that the underlying connective tissue facilitates and modulates epithelial growth and cellular differentiation [124,125].

If a material was to be implanted that degrades too quickly (>16 weeks), loss of volume would occur with poor or no tissue development [126]. When considering a material such as tetrapolymer PTFE-polyvinylidene fluoride (PVDF)-Polypropylene (PP) tetrapolymer or Artelon^®^ which breaks down over years [127], minimal tissue infiltration is observed and the regeneration of functional tissue (cellular interactions with the basement membrane vasculature, etc.), is not formed. Looking at the degradation of the Gesitlich Fibro-Gide^®^ material substitute [89], it is observed that after 3 months in vivo, tissue remodeling/healing is still occurring, with blood vessels formed in the material and some of the material still being present. However, the majority of material degradation has occurred [91], and thus, the degraded Fibro-Gide^®^ material has typically lost its physical form too early and presents properties on the lower end of what would be efficacious.

One way by which the mechanical properties of an electrospun scaffold can be modulated is by decreasing the material’s fiber diameter, which is typically proportional to an increased fiber density, providing greater mechanical stability. In a specific example using PCL/PEG fibers, it was found that as the proportion of PEG was increased, the mean fiber diameter decreased and the modulus of the material increased [118]. A Young’s modulus of 8.91MPa and 25–26MPa was determined with the PCL and PCL/PEG fibers, respectively [118]. Another related method for modulating electrospun fiber’s mechanical properties is through the use of sacrificial polymers. By using sacrificial polymers such as those described in the previous section [59,60], the fiber density and thus the mechanical properties can be adjusted, based on the fiber density of an electrospun membrane being proportional to the material’s Young’s modulus [128].

Previously, blends of defined ratios of PLA and PLGA have been fabricated [129]. In phosphate-buffered saline (PBS), PLGA and PLA scaffold degraded ~30% and ~15%, respectively with blends of the materials degrading 15–30% over 10 weeks [129]. This would then be expected to be a slower degradation rate compared to most natural scaffolds, however, the degradation product is lactic acid, which is known to interact with oral Streptococcus mutans (S. mutans), potentially leading to unfavorable outcomes such as S. mutans death, and changes in pH below that which is needed for the formation of caries [130]. In terms of mechanical properties, the pure PLGA and PLA scaffolds have a Young’s modulus from ~2 MPa to ~5 MPa, respectively, with the blends having moduli between 2 and 5 MPa [129]. Although the mechanical properties and degradation properties might be expected to be superior for gingival tissue regeneration to natural collagen-based scaffolds, the large immune response elicited by the materials, and their hydrophobic character are significant drawbacks, likely preventing the material’s adoption in the clinic [95]. Thus, the inferior biocompatibility must be offset with the partial substitution of natural polymers or the use of a new material entirely. Exploring electrospun blends of PCL and gelatin [131], PLA and gelatin [132], and many other blends of synthetic and natural polymers [103], have been previously reported on for tissue engineering and may offer promise for gingival tissue engineering [131].

As discussed in the previous section, the use of silk electrospun scaffolds with PDA and NH_2_-LIP shows promise. The addition of PDA and NH_2_-LIP to silk fibers increased the tensile strength from 1.95MPa to 2.87MPa [116]. Mechanical properties in this range seem reasonable considering native gingival tissue has a tensile strength of 3.8 MPa and buccal mucosa has a tensile strength of 1.5MPa [90]. Further, through a tissue engineering lens, we would expect the modulus of the materials to increase once seeded with cells [133]. Electrospun silk scaffolds have previously been shown to degrade in vivo within 8 weeks [134], which may be too quick to allow for the required anastomosis and tissue remodeling to occur, for the grafted volume to be retained and for the material to provide adequate efficacy. However, data do provide the support that vascularization of the silk fibers with PDA and NH_2_-LIP does occur in the oral cavity of a rabbit 14 days post-implantation [116]. A next step may be towards determining the in vivo efficacy of the material and/or pre-vascularized tissue constructs and comparing it to the Fibro-Gide^®^, Mucograft^®^, and Mucoderm^®^ materials. Determining the material’s suture pull-out strength could also be useful for evaluating the material’s handleability.

Another biomaterial that has been explored for the regeneration of several tissues, including gingival tissue, is segmented polyurethanes [135,136,137,138,139]. The soft and hard segments of these versatile biomaterials allow for control of the material’s mechanical and degradation properties. The degradation and mechanical properties can be used to strengthen and prolong the support provided for a scaffold by blending them with natural or other rapid-degrading polymers with weak mechanical characteristics. We have seen this achieved with some success in the treatment of wounds. For example, a blend of polyurethane and gelatin has previously been employed with the rationale of increasing degradation resistance and improving the mechanical properties of the material [135]. Using a 20% polyurethane and 80% gelatin material, in a collagenase/MMP-1 solution, the replacement of 20% gelatin for polyurethane resulted in the material degrading in 14 days versus within just 3 h in the 100% gelatin group [135]. While these specific formulations degrade too fast, there is much room for adjusting the ratios of natural polymers to polyurethanes, as well as the nature of the natural polymers and polyurethanes themselves. Further, investigators have previously suggested that efforts should be made to improve the degradation resistance of the Geistlich^®^ collagen-based matrices to enzymatic digestion, such as that from collagenase [43]. The addition of polyurethanes to natural scaffolds may offer the needed temporal degradation resistance to collagenase.

The efficacy of the material innovations that have been discussed here could be further improved through the addition of cells, which can be accomplished via a pre-seeding step to kick start the tissue formation process prior to implantation. The addition of cells to a material takes a step towards developing tissue in vitro which when used as a graft can decrease the time to engraftment [11], consequently leading to better efficacy.

## 8. Cell Options for Use with Electrospun Scaffolds

Previously, the application of pre-vascularized constructs for oral tissue grafting has been reviewed [11]. The review concluded that in vivo pre-vascularized implants had quicker integration with the host’s vasculature [11]. One main consideration when assessing engineering pre-vascularized constructs is the cell source itself. During periodontal tissue regeneration, HGFs play a fundamental role in establishing the needed ECM required to achieve integration between the relevant tissues [33] and support ECs that contribute to enabling angiogenesis [140]. Thus, a co-culture of ECs and support cells such as fibroblast-like cells, and vice-versa, should provide the required biological cues for the formation of vasculature and gingival tissue regeneration [141]. 

With the aim of regenerating the buccal tissue, a triculture with epithelial cells was seeded on one side of either a Geistlich Bio-Gide^®^ or Bio-Gide^®^ Pro (further crosslinked) scaffold with fibroblasts from the gingiva, and microvascular endothelial cells from human juvenile foreskin (in a 1:1 ratio) being seeded on the other side [142]. After 10 days of subcutaneous implantation in a mouse, the seeded pre-vascularized constructs had evidence of red blood cells present within the constructs. Based on these findings and the previously mentioned review [11], the efficacy and shift towards the use of pre-vascularized constructs appear to be occurring. The use of clinically convenient and practically sourced cells to be obtained from the patient to allow for the generation of an autologous graft still appears to remain a challenge.

The use of adipose tissue may be of particular interest as both fibroblast-like human adipose-derived stem cells (ASCs) and human adipose-derived microvascular endothelial cells (HAMVECs) can be obtained from a single tissue sample [143]. ASCs are a heterogeneous group of fibroblast-like cells [144], which are phenotypically indistinguishable from fibroblasts [145]. Only 5% of genes are uniquely expressed between HGFs and human dermal fibroblasts, with their fundamental characteristics being the same [146]. This suggests that gingival-specific fibroblasts may not be necessary for gingival tissue grafting. However, a functional comparison is lacking in the literature. Adipose tissue may provide an inexpensive, practical, and autologous source of fibroblast-like cells and ECs.

Due to the reciprocal interaction of the soft tissue underlying the basement membrane and keratinized tissue, the use of a cell type that can either form a basement membrane or an epithelium would be expected to be advantageous for the support of keratinized tissue. More specifically, it is beta1-integrins and other integrins found in the ECM of the underlying connective tissue that regulates and dictate cellular differentiation, detachment, apoptosis, and other cellular behaviors [38]. Previously, ASCs have been shown to produce an epithelium with a basement membrane not fully developed compared to a culture of HGFs [124]. ASCs have also been shown to be able to differentiate into keratinocytes [147]. Adipose tissue appears to offer a practical and convenient source of microvasculature cells, fibroblast-like cells, and keratinocytes [147], which accounts for the majority of the cellular components comprising gingival tissue [13,17]. 

Determining the efficacy of the novel acellular materials and cell-seeded materials discussed here will also need to be accompanied by the development of new pre-clinical animal models. There are recent reports on the use of an oral defect rabbit model, however, the latter model does have limitations, including the observation that the experiment was only carried out 14 days post-operation [116]. With the rabbit defect model being reported in 2020 the field will likely continue to build and improve on the model. Pre-clinical models for oral reconstructive therapies have previously been reviewed [148], with no reports of rodent animal models specifically targeted for evaluating the efficacy of tissue-engineered implants for gingival recession. Such consideration is an area of critical development needed for early pre-clinical assessment in the future. This will contribute to advancing the new materials and specifically cell-seeded constructs, before moving to larger animal models which are more costly, and ultimately clinical trials.

It should be noted that a limitation of this review is the lack of in vivo data directly comparing the novel materials and electrospun layered constructs to the family of Geistlich materials (Fibro-Gide^®^, Mucograft^®^, and Mucoderm^®^). Hence, this represents an area of future growth. Having said this, there is in vivo evidence that multilayered electrospun constructs facilitate the formation of vascularized tissue [82], and we are beginning to see gingival-specific pre-clinical animal models. The field will now need to work towards evaluating novel constructs using gingival-specific in vivo models and compare them to the current material standards.

## 9. Conclusions

With the field of gingival tissue engineering being in its infancy, the use of layered electrospun composite materials appears to offer the versatility needed to generate structured materials and constructs with function towards the regeneration of gingival tissue. The literature offers a number of specific strategies for modulating a scaffold’s function that can be used to optimize materials for the treatment of gingival recession. Specifically, significant work is being directed towards blended synthetic/natural material substitutes and autologous pre-vascularized cell-seeded grafts. A material with multiple optimized functional design features, such as degradation, porosity, potential to vascularize, and loading with bioactive molecules, is likely to have the most promise for clinical success. Future work will need to focus on evaluating the ability of the prepared materials or constructs to support the development of healthy functional gingival tissue and its relevant cells.

## Figures and Tables

**Table 1:** An outline of the vessels found within gingival tissue adapted from previous literature [24].

Tissue Area	Type of Vessel	Diameter (µm)	Average Depth (µm)
Free gingiva	Capillary loops	≤30	50–200
	Connective vessels	50–100	200–700
	Large blood vessels	200–400	≥500
Attached gingiva	Capillary loops	≤15	50–200
	Connective vessels	-	-
	Large blood vessels	200–500	≥600
Alveolar mucosa	Capillary loops	≤15	50–200
	Connective vessels	200–600	200–700
	Large blood vessels	≥600	≥700

## Data Availability

Not applicable.
